# Pneumothorax: Demographics, Treatment, and Nursing Care

**DOI:** 10.3390/healthcare14131901

**Published:** 2026-06-30

**Authors:** Ivana Herak, Mirna Korpar, Sonja Obranić, Mario Gašić, Anita Lukic

**Affiliations:** 1 Department of Nursing, University North, 104 Brigade 3, 42000 Varazdin, Croatia; ivherak@unin.hr (I.H.); mkorpar00@gmail.com (M.K.); sobranic@unin.hr (S.O.); 2 Varaždin General Hospital, 1 I Mestrovica Street, 42000 Varazdin, Croatia; 3 Emergency Medicine Center OHBP General Hospital, Bana Jelačića 10, 31500 Nasice, Croatia; 7digital1986@gmail.com; 4 Department of Nursing, Bjelovar University of Applied Sciences, 4 E Kvaternika Square, 43000 Bjelovar, Croatia

**Keywords:** pneumothorax, spontaneous pneumothorax, traumatic pneumothorax, iatrogenic pneumothorax, nursing care, NANDA-I, nursing dependency, chest drainage

## Abstract

**Highlights:**

**What are the main findings?**

- In a three-year single-centre cohort of 60 hospitalised patients, pneumothorax occurred predominantly in older men, with chest drainage used in 73.3% (95% CI 61.0–82.9) of admissions, and in all iatrogenic cases.
- An exploratory L1-penalised multivariable model identified age ≥60 years as the only covariate independently associated with the receipt of chest drainage (adjusted OR 3.67; 95% CI 1.21–13.56; *p* = 0.026); dependency at admission and sex were not independent predictors.

**What are the implications of the main findings?**

- The substantial nursing-care workload—a mean of 1.93 NANDA-I diagnoses per patient and functional deterioration in 23.3% of admissions—underlines the value of integrating nursing dependency and risk-oriented diagnoses into pneumothorax care planning.
- These descriptive findings provide a baseline for the Croatian setting and generate testable hypotheses for larger, prospective, multicentre studies that combine clinical and nursing-sensitive endpoints.

**Abstract:**

Background: Pneumothorax is a clinically heterogeneous condition with a substantial nursing-care burden; yet, contemporary descriptive data that combine medical and nursing variables remain scarce in the Croatian setting. Our aim was to describe the demographic profile, treatment patterns, and nursing-care requirements of patients treated for pneumothorax at a single regional hospital. Moreover, we explored factors associated with chest drainage and with adverse nursing-sensitive outcomes. Methods: We conducted a retrospective, single-centre, cross-sectional analysis of adult and adolescent patients consecutively admitted due to pneumothorax to Varaždin General Hospital between 1 January 2019 and 31 December 2021. Sociodemographic, clinical, and nursing-care variables were extracted from the hospital information system and the electronic nursing documentation. Nursing diagnoses were classified using NANDA International (NANDA-I) terminology. Proportions are reported with 95% Wilson score confidence intervals. Bivariate associations between categorical variables were assessed using the Fisher exact test with Haldane–Anscombe-corrected odds ratios; the Kruskal–Wallis test with Bonferroni-corrected pairwise comparisons were used for continuous distributions. Independent associations with the chest drainage placement, prolonged length of stay (>14 days), and worsening of dependency category were assessed with L1-penalised logistic regression (α = 0.5), with 1000-iteration non-parametric bootstrap 95% CIs and *p*-values. Results: Of 60 patients included, 39 (65.0%; 95% CI 52.4–75.8) were male and 33 (55.0%; 42.5–66.9) were aged 60 years or older. Spontaneous pneumothorax accounted for 27 cases (45.0%; 33.1–57.5), traumatic for 23 (38.3%; 27.1–51.0), and iatrogenic for 10 (16.7%; 9.3–28.0). Chest drainage was used in 44 patients (73.3%; 61.0–82.9), universally in iatrogenic cases. After adjustment, age ≥ 60 years was independently associated with the receipt of chest drainage (adjusted OR 3.67; 95% CI 1.21–13.56; *p* = 0.026), with a prolonged length of stay (adjusted OR 3.69; 95% CI 1.02–21.00; *p* = 0.042) and with functional deterioration (adjusted OR 4.29; 95% CI 1.21–22.62; *p* = 0.028). Risk for falls (58.3%) and Bathing self-care deficit (26.7%) were the most frequent NANDA-I diagnoses; 14 patients (23.3%) deteriorated by at least one dependency category by discharge. Conclusions: Patients hospitalised with pneumothorax at our centre were predominantly older men with a substantial nursing-care workload. An older age was the most consistent independent correlate of both invasive treatment and adverse nursing-sensitive outcomes. The findings provide a descriptive baseline for the Croatian setting and should be interpreted as hypothesis-generating, given the modest sample size and the single-centre retrospective design.

## 1. Introduction

Pneumothorax is defined as the presence of air in the pleural space, resulting in the partial or complete collapse of the underlying lung [1,2]. It is conventionally classified according to the mechanism of the air entry into the pleural cavity. Spontaneous pneumothorax occurs in the absence of preceding trauma or medical intervention. It is further subdivided into primary spontaneous pneumothorax, occurring in patients without clinically apparent lung disease, and secondary spontaneous pneumothorax, occurring on the background of an underlying pulmonary disorder such as chronic obstructive pulmonary disease (COPD), interstitial lung disease, or pulmonary infection [1,3,4]. On the other hand, traumatic pneumothorax results from blunt or penetrating injury to the chest wall, whereas iatrogenic pneumothorax is a complication of diagnostic or therapeutic procedures, including transthoracic biopsy, central venous catheter insertion, mechanical ventilation, and thoracentesis [3,5,6]. Tension pneumothorax represents a clinical emergency that may complicate any of the above subtypes and requires immediate decompression [7].

The epidemiological data on pneumothorax vary considerably across geographical regions and over time. The reported incidence rates for primary spontaneous pneumothorax range from approximately 7 to 18 cases per 100,000 population per year in men and from 1 to 6 cases per 100,000 in women, with a male-to-female ratio of approximately 3:1 to 6:1 [1,7,8]. More recent population-based reports have additionally highlighted pneumothorax as a recognised complication of viral pneumonia and mechanical ventilation in critically ill patients, particularly during and after the COVID-19 pandemic [4,9,10]. The age distribution is characteristically bimodal, with a first peak in young adults aged 15–34 years and a second peak in patients over 55 years, the latter being predominantly accounted for by secondary spontaneous and iatrogenic cases [1,11]. Established risk factors include cigarette smoking, a low body mass index, a tall and lean body habitus, a positive family history, and the presence of underlying lung disease, particularly COPD [1,3,6]. However, meteorological and microclimatic factors do not appear to play a substantial role in onset [12].

Although primary spontaneous pneumothorax is generally a benign condition, secondary spontaneous and traumatic pneumothoraxes may be associated with substantial morbidity, prolonged hospitalisation, and a measurable mortality risk [8,13]. The treatment of pneumothorax depends on the size of the air collection, the presence and severity of symptoms, the aetiological category, and the patient’s overall clinical state.

Contemporary guidelines and major reviews increasingly endorse a graded approach. This approach based on conservative management (clinical observation with supplemental oxygen) is appropriate for selected small, minimally symptomatic pneumothoraxes, particularly of primary spontaneous origin [7,14,15]. Larger or symptomatic pneumothoraxes are most commonly managed with tube thoracostomy (chest drainage), which remains the most widely used invasive intervention across European centres and constitutes the current standard of practice in our institution [7,14,16]. Alternative approaches include needle aspiration and small-bore catheter drainage, including ambulatory devices with one-way valves, which have shown a comparable efficacy and shorter length of stay in selected populations [16,17]; surgical interventions (video-assisted thoracoscopic surgery or open thoracotomy) are reserved for a persistent air leak or recurrent pneumothorax [7,14]. Importantly, a recent open-label randomised trial demonstrated that conservative management was not inferior to interventional management for moderate-to-large primary spontaneous pneumothorax, with a lower rate of serious adverse events [18]. Comparative evidence from the trauma setting suggests broadly similar outcomes between small-bore pigtail catheters and standard chest tubes [19]. The choice between these options is therefore influenced by clinical guidelines, the local availability of equipment and expertise, and patient preference.

Although the medical management of pneumothorax has been extensively described, less attention has been paid to the nursing-care burden in these patients. Patients with pneumothorax, particularly those undergoing prolonged chest drainage, typically require a structured assessment of risks related to falls, pressure ulcer development, infection, and self-care deficits, as well as the continuous monitoring of vital signs, drain function, and the integrity of the underwater-seal or one-way-valve system [20,21]. A recent scoping review of nursing care for adults with chest drainage identified surveillance of the drainage system, prevention of infection, early mobilisation, and structured patient education as the most consistently reported nursing responsibilities, and highlighted the considerable variability in how these activities are documented across institutions [20]. The documentation of these requirements using standardised terminologies such as NANDA International (NANDA-I) [22] facilitates comparability across institutions and supports evidence-based nursing practice; yet, such systematic descriptions remain uncommon in the pneumothorax literature, particularly in central and south-eastern Europe.

In that light, our aim was to describe the sociodemographic, clinical, and nursing-care characteristics of patients treated for pneumothorax at a regional Croatian hospital over a three-year period.

In addition, we explored several research questions based on the available data. The primary question was whether patients aged 60 years or older were more likely to require chest drainage after adjustment for sex and nursing-care dependency. We also examined whether these same factors were associated with a prolonged hospital stay (>14 days) and with worsening nursing-care dependency between admission and discharge. Our null hypothesis was that age, sex, and nursing-care dependency were not associated with any of these outcomes. The alternative hypothesis was that at least one of these factors, particularly age ≥ 60 years, was associated with the outcomes of interest.

Because this was a descriptive single-centre study with a relatively small sample, the analyses were exploratory and intended to identify potential associations rather than establish causal relationships.

## 2. Materials and Methods

### 2.1. Study Design and Setting

This was a retrospective, single-centre, cross-sectional study based on routinely collected clinical and nursing-care data. The study was conducted at Varaždin General Hospital, a regional secondary-care hospital serving Varaždin County and the surrounding area in north-western Croatia. The hospital provides comprehensive emergency, medical, and surgical care, including the management of thoracic emergencies, and is the principal hospital responsible for the admission and treatment of patients with pneumothorax in the region. The study period was from 1 January 2019 to 31 December 2021, inclusive. The manuscript is reported in accordance with the relevant items of the STROBE statement for cross-sectional studies.

### 2.2. Ethical Considerations

The study was conducted in accordance with the Declaration of Helsinki and was approved by the Ethics Committee of Varaždin General Hospital (approval No. 02/1-91/108-2022, dated 13 May 2022). Because the study used only retrospectively collected, de-identified data extracted from existing clinical and nursing records, the requirement for individual informed consent was waived by the Ethics Committee. No patient identifiers were extracted or stored, and all data were handled in accordance with the institution’s data-protection policies and with applicable national and European legislation on the protection of personal data, including the General Data Protection Regulation.

### 2.3. Study Population and Eligibility Criteria

All patients admitted to Varaždin General Hospital during the study period with a primary discharge diagnosis of pneumothorax (International Classification of Diseases, 10th revision, code J93) were eligible for inclusion. Inclusion criteria were (i) admission and discharge from Varaždin General Hospital during the defined study period, and (ii) availability of complete medical and nursing-care documentation in the electronic hospital information system. The single exclusion criterion was transfer to another institution during the index admission, which would have resulted in incomplete capture of outcomes. A total of 60 patients met the eligibility criteria and were included in the analysis. Because the entire eligible source population during the three-year period was enumerated, no further sampling was undertaken.

### 2.4. Variables and Definitions

For each patient, the following data were collected: sex, age, type of pneumothorax, treatment approach, duration of chest drainage, length of hospital stay, nursing-care dependency at admission and discharge, and documented nursing diagnoses.

Age was initially recorded in six categories (<18, 18–29, 30–39, 40–49, 50–59, and ≥60 years) according to the existing data-collection form. For inferential analyses, age was additionally grouped into two categories (<60 and ≥60 years).

Pneumothorax was classified as spontaneous, traumatic, or iatrogenic based on the discharge summary. Treatment was categorised as either chest drainage or conservative management.

The duration of chest drainage was grouped into three categories (1–7 days, 8–14 days, and >14 days). A separate category was used for patients who were transferred to another hospital while the drain was still in place, making the total drainage duration unavailable.

Length of hospital stay was also categorised as 1–7 days, 8–14 days, or >14 days. For inferential analyses, hospital stay was further dichotomised as prolonged (>14 days) or non-prolonged (≤14 days).

Nursing-care dependency was recorded both at admission and discharge, allowing assessment of changes during hospitalisation.

Nursing-care dependency was assessed using the four-tier patient classification system routinely applied in Croatian hospitals. It is based on structured assessment of activities of daily living, including personal hygiene, feeding, mobility, and elimination, as well as monitoring requirements and the complexity of nursing interventions. In this system, Category 1 represents the least dependent patients who are largely capable of self-care, whereas Category 4 represents the most dependent patients who require extensive nursing support. The assessment using this classification was performed at admission and repeated at discharge by the ward nurse. For inferential analyses, nursing-care dependency was additionally grouped into low dependency (Categories 1–2) and high dependency (Categories 3–4). Although developed for routine clinical practice, the Croatian four-tier classification system is conceptually comparable to patient-classification systems used internationally in studies of nursing workload and patient care needs [23,24,25].

Nursing diagnoses recorded in the electronic nursing documentation were classified retrospectively according to the NANDA International (NANDA-I) Nursing Diagnoses: Definitions and Classification, 2024–2026 edition [22]. Classification was performed by a senior nurse (I.H.) with prior formal training in NANDA-I terminology, while ambiguous cases were resolved by consensus with a second rater. For each patient, all diagnoses recorded during the hospital stay were included, and a single patient could therefore contribute more than one diagnosis. Patients in whom no nursing diagnosis was documented were reported separately rather than as a diagnostic category.

Three nursing-sensitive outcomes were pre-specified for the inferential analyses: receipt of chest drainage (yes/no), prolonged length of hospital stay (>14 days vs. ≤14 days), and worsening of the dependency category between admission and discharge (any increase by at least one category vs. no change or decrease).

Conservative management was defined as supportive care with no invasive drainage of the pleural space (typically clinical observation with supplemental oxygen), while chest drainage was defined as the placement of a chest tube connected to either an underwater-seal system, a one-way valve, or active suction [7,14].

### 2.5. Data Sources and Data Collection

Data were extracted from the hospital information system (WinBis, version 214, 2026, In2 group Zagreb, Croatia) and from the electronic nursing documentation by a single trained investigator (M.K.) using a structured extraction template developed for this study. The template included pre-coded fields for each variable described in Section 2.4. To minimise extraction error, a 10% random sample of records was independently re-extracted by a second investigator and compared with the primary extraction; agreement exceeded 95% for all variables. During the preparation of the present manuscript, all extracted values were systematically re-verified against the original hospital documentation by two of the authors (I.H. and A.L.), and a small number of transcription errors in the original nursing-diagnosis frequency table were corrected. Where applicable, the labelling of nursing diagnoses was harmonised with the NANDA-I 2024–2026 terminology [22].

### 2.6. Sample Size and Statistical Analysis

Because this was a descriptive study that included all eligible patients treated during the three-year study period, the sample size of 60 patients was determined and limited by the number of available cases rather than by predefined sample size calculation.

To assess the implications of the sample size, a post hoc power analysis was performed using G*Power (version 3.1.9.7, Heinrich Heine University Düsseldorf, Düsseldorf, Germany) [26]. With 60 patients, a two-sided significance level of 0.05, and a statistical power of 80%, the study was able to detect associations corresponding to a Cramér’s V of approximately 0.36 in a χ^2^ test of independence for a 2 × 2 contingency table, which is generally considered a large effect size according to Cohen’s criteria. Consequently, the study had limited ability to detect small or moderate associations. Findings that did not reach statistical significance should therefore be interpreted with caution, particularly in the multivariable analyses where the number of outcome events per variable was limited.

Continuous variables are summarised as median and interquartile range (IQR); categorical variables are summarised as absolute frequencies and percentages. For all proportions, 95% confidence intervals were calculated using the Wilson score method, which provides more reliable estimates than the normal approximation, particularly in moderately sized samples [27].

Associations between categorical variables were analysed using Fisher exact test for 2 × 2 contingency tables. Odds ratios and their 95% confidence intervals were calculated using the Haldane–Anscombe continuity correction (addition of 0.5 to each cell) to avoid unstable estimates when zero cell counts were present. For larger contingency tables with more than two categories on one or both axes (r × c), the Fisher–Freeman–Halton exact test was applied using a Monte Carlo approximation with 10,000 iterations [28,29].

The differences in the length of hospital stay among the three pneumothorax types were analysed using the Kruskal–Wallis’s test. When a significant difference was identified, post hoc pairwise comparisons were performed using the Mann–Whitney U test with Bonferroni correction for multiple testing. Length of hospital stay was represented by the midpoint of the corresponding categorical interval.

The changes in nursing-care dependency between admission and discharge was assessed using the Wilcoxon signed-rank test. Effect sizes for categorical comparisons are reported as Cramér’s V.

To explore independent associations between sociodemographic and care-related factors and the three pre-specified nursing-sensitive outcomes (placement of chest drainage, prolonged length of stay, and worsening of dependency category), we fitted L1-penalised (lasso-type) logistic regression models with a penalty strength of α = 0.5.

Penalised logistic regression was chosen over conventional unpenalised maximum-likelihood estimation because of the modest sample size and the limited number of events per variable, particularly in the prolonged length-of-stay and dependency-deterioration models (3.2 and 3.5 events per, respectively). Under such conditions, conventional maximum-likelihood logistic regression may produce unstable and biased estimates [30,31]. For each model, adjusted odds ratios (aORs), 95% confidence intervals, and two-sided *p*-values were estimated using 1000 non-parametric bootstrap replications of the original dataset.

Candidate covariates were selected a priori on clinical grounds and included age ≥60 years, male sex, and high nursing dependency (Categories 3–4) at admission. Where appropriate, chest drainage and prolonged length of stay were also included as covariates. Pneumothorax type was not entered into the model for receipt of chest drainage because all iatrogenic pneumothoraxes were treated with drainage, which resulted in complete separation.

Given the modest sample size and the exploratory nature of the study, these adjusted estimates should be interpreted as hypothesis-generating rather than as definitive evidence of causal associations.

All statistical tests were two-sided, and statistical significance was defined a priori as *p* < 0.05. *p*-values below 0.001 are reported as *p* < 0.001.

With the exception of the post hoc power analysis mentioned above, all analyses were performed in IBM SPSS Statistics for Windows, version 30.0 (IBM Corp., Armonk, NY, USA). Python (version 3.11; SciPy 1.11 and scikit-learn 1.4) was used as a complementary platform for calculating the Wilson confidence intervals, the Fisher exact test with Haldane–Anscombe-corrected odds ratios, the L1-penalised logistic regression models, and the bootstrap-based estimates of confidence intervals and *p*-values.

## 3. Results

### 3.1. Sociodemographic and Clinical Characteristics

A total of 60 patients met the eligibility criteria during the three-year study period and were included in the analysis. Men accounted for 39 patients (65.0%; 95% CI 52.4–75.8) and women for 21 (35.0%). The age distribution was skewed towards older age groups: 33 patients (55.0%; 42.5–66.9) were aged 60 years or older, while 12 (20.0%) were in the youngest adult band (18–29 years), reflecting the characteristic bimodal age distribution previously described for hospitalised pneumothorax [1,11]. The categorical age distribution is summarised in Table 1.

Spontaneous pneumothorax was the most common (27 patients; 45.0%; 95% CI 33.1–57.5), followed by traumatic (23; 38.3%; 27.1–51.0) and iatrogenic (10; 16.7%; 9.3–28.0) (Table 2).

Chest drainage was the predominant treatment modality, used in 44 patients (73.3%; 61.0–82.9); the remaining 16 (26.7%) were managed conservatively. The drain was in place for 1–7 days in 21 patients (47.7%), for 8–14 days in 15 (34.1%), and for more than 14 days in 2 (4.5%); the drainage duration could not be determined for 6 patients (13.6%) transferred to another institution while the drain was in situ.

**Figure 1:**
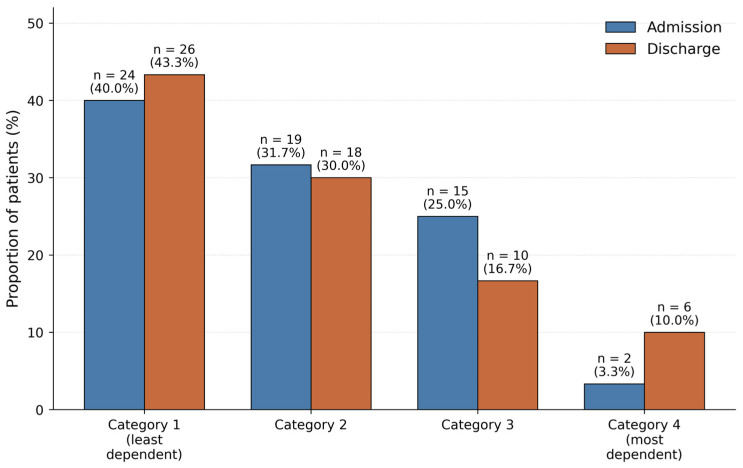
Distribution of nursing-care dependency at admission and at discharge (n = 60). Bars give the number of patients in each category, with the corresponding percentage of the study population shown above each bar. The proportion of patients in Category 4 increased from 3.3% at admission to 10.0% at discharge, although the overall change in dependency category did not reach statistical significance (Wilcoxon signed-rank test, *p* = 0.866).

The overall length of hospital stay was 1–7 days in 25 patients (41.7%), 8–14 days in 22 (36.7%), and more than 14 days in 13 (21.7%; 13.1–33.6) (Figure 1).

Nursing-care dependency at admission was distributed across all four categories: 24 patients (40.0%) were classified as Category 1 (least dependent), 19 (31.7%) as Category 2, 15 (25.0%) as Category 3, and 2 (3.3%) as Category 4 (most dependent), so that 17 patients (28.3%; 95% CI 18.5–40.8) were in the high-dependency stratum (Categories 3–4). By discharge, the distribution had shifted only modestly: 26 patients (43.3%) were in Category 1, 18 (30.0%) in Category 2, 10 (16.7%) in Category 3, and 6 (10.0%) in Category 4 (Figure 1). Overall, the change in dependency category between admission and discharge did not reach statistical significance (Wilcoxon signed-rank test, *p* = 0.866). Nevertheless, 14 patients (23.3%; 14.4–35.4) were assigned to a higher dependency category at discharge than at admission, indicating functional deterioration during the hospital stay. The paired distribution of dependency categories at admission and at discharge is shown graphically in Figure 1, which makes visible the three-fold rise in the proportion of patients in the most-dependent Category 4 (from 3.3% to 10.0%) that is otherwise obscured by the non-significant overall shift. The pooled descriptive characteristics with their 95% Wilson confidence intervals are additionally presented in Table 2 for ease of cross-referencing in the subsequent inferential analyses.

### 3.2. Associations with Type of Pneumothorax, Treatment Modality, and Length of Stay

Bivariate associations between sociodemographic characteristics and the type of pneumothorax and treatment modality were re-examined after the re-verification of the original hospital documentation. A small number of transcription errors in the originally tabulated data was found and the data were corrected. No significant association was observed between sex and the type of pneumothorax (Fisher exact *p* = 0.594). In contrast, age group (dichotomised at 60 years) was significantly associated with the type of pneumothorax (Fisher–Freeman–Halton *p* = 0.019; Cramér’s V = 0.42, indicating a moderate effect): older patients accounted for 65.2% of traumatic and 80.0% of iatrogenic cases, compared with 37.0% of spontaneous cases.

The length of the hospital stay did not differ significantly between spontaneous, traumatic, and iatrogenic pneumothorax. The median length of stay was 11 days in all three groups, although patients with iatrogenic pneumothorax tended to have a wider distribution of hospital stay durations (Kruskal–Wallis H = 3.37; *p* = 0.185). Pairwise post hoc Mann–Whitney comparisons between pneumothorax types were also non-significant after Bonferroni correction (spontaneous vs. traumatic, Pcorr = 1.000; spontaneous vs. iatrogenic, Pcorr = 0.209; and traumatic vs. iatrogenic, Pcorr = 0.527).

Prolonged hospital stay (>14 days) was observed most frequently among patients with iatrogenic pneumothorax (40.0%; 95% CI 16.8–68.7) and least frequently among those with spontaneous pneumothorax (14.8%; 95% CI 5.9–32.5), with traumatic pneumothorax showing intermediate values (21.7%; 95% CI 9.7–41.9). However, these differences did not reach statistical significance (Fisher–Freeman–Halton *p* = 0.462) (Table 3).

The treatment modality was not significantly associated with either sex (Fisher exact *p* = 0.064) or age group (χ^2^ *p* = 0.206). In contrast, treatment modality differed significantly according to pneumothorax type (Fisher–Freeman–Halton *p* = 0.013). All patients with iatrogenic pneumothorax underwent chest drainage, compared with 78.3% of patients with traumatic pneumothorax and 59.3% of those with spontaneous pneumothorax.

After verification of the dependency data, the previously observed association between nursing-care dependency at admission and treatment modality was no longer present when dependency was grouped into clinically meaningful categories of low (Categories 1–2) and high (Categories 3–4) dependency. Chest drainage was performed in 10 of 17 high-dependency patients (58.8%) and 34 of 43 low-dependency patients (79.1%) (Fisher exact OR 0.39; 95% CI 0.12–1.26; *p* = 0.193).

Although the analysis of the original four-category scale produced a nominally significant result (χ^2^ = 8.45; *p* = 0.038), more than one-third of cells (37.5%) had expected frequencies below 5. Given these sparse data, the Fisher exact analysis based on the dichotomised dependency scale was considered more robust and is reported as the primary inferential estimate.

Bivariate associations of pre-specified covariates with a prolonged length of stay (>14 days) and with a worsening of the dependency category are summarised in Table 4 and Table 5. Age ≥ 60 years was the only covariate significantly associated with a prolonged length of stay (OR 5.21; 95% CI 1.19–22.91; *p* = 0.026) and with the deterioration of dependency category (OR 5.93; 95% CI 1.36–25.90; *p* = 0.013).

To identify factors independently associated with the three nursing-sensitive outcomes, L1-penalised logistic regression models (α = 0.5) with 1000-bootstrap confidence intervals and *p*-values were fitted (Table 5).

Across all three models, age ≥ 60 years emerged as the only factor consistently associated with the outcomes. An older age was independently associated with the receipt of chest drainage (adjusted OR 3.67; 95% CI 1.21–13.56; *p* = 0.026), a prolonged length of stay (adjusted OR 3.69; 95% CI 1.02–21.00; *p* = 0.042), and a worsening of nursing-care dependency during hospitalisation (adjusted OR 4.29; 95% CI 1.21–22.62; *p* = 0.028). In the chest drainage model (n = 60; 44 events), the male sex showed a non-significant trend toward an increased likelihood of drainage (adjusted OR 2.82; 95% CI 1.00–10.05; *p* = 0.100), whereas a high nursing dependency at admission was not independently associated with treatment modality (adjusted OR 0.46; 95% CI 0.10–1.06; *p* = 0.240). In the prolonged length-of-stay model (n = 60; 13 events), neither male sex, chest drainage, nor high nursing dependency retained an independent association after adjustment. Similarly, in the model for the worsening of nursing-care dependency (n = 60; 14 events), male sex, chest drainage, and prolonged length of stay were not independently associated with deterioration after adjustment.

### 3.3. Nursing Diagnoses

A nursing diagnosis was documented for 56 of the 60 patients (93.3%); the remaining 4 (6.7%) had no diagnosis recorded in the electronic nursing documentation. A total of 116 individual diagnostic episodes were recorded across the cohort, corresponding to a mean of 1.93 NANDA-I diagnoses per patient (standard deviation 1.25; median 2; IQR 1–3; range 0–6). The frequency of all documented NANDA-I diagnoses, with their 95% Wilson confidence intervals, is presented in Table 6.

Associations between the five most prevalent NANDA-I nursing diagnoses and the pre-specified covariates are presented in Table 7.

The strongest associations were observed for Risk for falls and Risk for pressure ulcers. Patients aged ≥60 years were more likely to have a diagnosis of Risk for falls than younger patients (75.8% vs. 37.0%; OR 5.15; 95% CI 1.54–18.91; *p* = 0.004). The diagnosis was also more common among patients whose nursing-care dependency worsened during hospitalisation (85.7% vs. 50.0%; OR 5.84; 95% CI 1.11–59.53; *p* = 0.028).

Similarly, Risk for pressure ulcers was more frequent among patients with a prolonged hospital stay (46.2% vs. 10.6%; OR 6.87; 95% CI 1.35–37.97; *p* = 0.009) and among those with high nursing dependency at admission (41.2% vs. 9.3%; OR 6.55; 95% CI 1.36–37.07; *p* = 0.008).

No statistically significant associations were identified between the remaining prevalent NANDA-I diagnoses and the pre-specified covariates.

At the patient level, the number of recorded nursing diagnoses was higher among patients with greater care needs. Patients aged ≥ 60 years had more nursing diagnoses than younger patients (median 2 [IQR 1–3] vs. 1 [IQR 1–2]; *p* = 0.006), and patients with a prolonged hospital stay also had more nursing diagnoses than those with shorter stays (median 2 [IQR 2–3] vs. 1 [IQR 1–2]; *p* = 0.001).

Similarly, patients with a high nursing-care dependency at admission and those whose dependency category worsened during hospitalisation had significantly more nursing diagnoses recorded than their counterparts (both *p* = 0.048).

In contrast, the number of nursing diagnoses did not differ significantly according to sex or pneumothorax type (*p* = 0.424 and *p* = 0.662, respectively). However, a significant difference was observed across the four admission dependency categories (*p* = 0.015) (Table 8).

## 4. Discussion

This retrospective single-centre study describes the demographic profile, treatment patterns, and nursing-care requirements of patients hospitalised with pneumothorax at Varaždin General Hospital over a three-year period. Three observations stand out. First, the patient population was older and more strongly male-predominant than is generally reported for primary spontaneous pneumothorax, reflecting the mixed aetiological composition of admissions to a regional secondary-care hospital. Second, chest drainage was the predominant treatment modality and was applied uniformly in iatrogenic cases; after adjustment, only age ≥ 60 years was independently associated with its receipt. Third, the cohort generated a substantial and clinically meaningful nursing-care workload, with a high prevalence of risk-oriented NANDA-I diagnoses, an average of nearly two diagnoses per patient and functional deterioration in almost one in four admissions.

In other words, the findings suggest that patient age and nursing-care dependency are more important determinants of nursing workload and clinical complexity than sex or pneumothorax type. Patients aged 60 years and older were consistently more likely to experience adverse outcomes, including the need for chest drainage, prolonged hospitalisation, and worsening nursing-care dependency. Similarly, a higher dependency at admission and the deterioration of dependency during hospitalisation were associated with greater nursing complexity, reflected in a higher number of documented nursing diagnoses. A prolonged hospital stay was likewise linked to increased nursing burden, particularly through a greater prevalence of nursing diagnoses and pressure-ulcer risk. In contrast, although some differences were observed in the descriptive analyses, the sex and pneumothorax type showed limited and inconsistent associations with the outcomes once other factors were taken into account.

### 4.1. Demographic Characteristics

The age and sex distribution observed in our cohort is consistent with the epidemiological data for the broader hospitalised pneumothorax population, in which a substantial proportion of admissions are accounted for by secondary spontaneous and traumatic cases in older men [3,5,6,8,35]. The reported population-level incidence of pneumothorax in high-income European settings is broadly comparable to the case-mix we observed, although direct comparisons should be made with caution given the differences in case ascertainment and the inclusion of iatrogenic pneumothorax [1,5,7,8]. Our finding that patients aged 60 years or older accounted for the majority of the cohort and were over-represented in the traumatic and iatrogenic categories is in keeping with the recently described secular trend towards an older pneumothorax population in countries with ageing demographics [8].

The aetiological composition of our cohort—spontaneous (45.0%), traumatic (38.3%), and iatrogenic (16.7%)—differs from cohorts focused on primary spontaneous pneumothorax in younger patients, but is broadly aligned with population-based studies that include all pneumothorax admissions [6,8,35]. Our relatively high proportion of iatrogenic cases (16.7%) is consistent with reports from contemporary tertiary settings, where the greater use of invasive diagnostic and therapeutic procedures has been associated with a rising iatrogenic incidence over time [11,13]. The numerically longer length of stay in iatrogenic cases, although not statistically significant on Kruskal–Wallis’s testing, is in keeping with reports that iatrogenic pneumothorax tends to occur in patients with greater baseline morbidity [11].

### 4.2. Treatment Patterns and Contextual Interpretation

Our observed treatment distribution—73.3% chest drainage and 26.7% conservative management—reflects the local practice during the study period and is consistent with current European recommendations, in which tube thoracostomy remains the most widely applied invasive intervention for larger or symptomatic pneumothoraxes [7,14,15,17]. The recently revised guidelines from the French societies for pulmonology, emergency medicine, intensive care, anaesthesia, and thoracic surgery emphasise a stepwise, individualised approach in which the size of the pneumothorax, the severity of symptoms, the underlying aetiology, and the patient’s comorbidity profile jointly inform the choice between observation, needle aspiration, small-bore catheter drainage, and standard chest-tube placement [14]. Comparable recommendations have been articulated in the contemporary British Thoracic Society framework and in narrative reviews of modern pneumothorax management [15,16]. Our data should therefore be interpreted as describing the practice pattern of a single regional centre during a defined period; they should not be construed as evidence that chest drainage is the preferred or optimal first-line treatment across all pneumothorax categories.

Importantly, the bivariate associations between the sociodemographic variables and treatment modality observed in our cohort must be interpreted with substantial caution. The recent randomised evidence has called into question the previous assumption that intervention is universally required for large primary spontaneous pneumothorax: in the PSP trial, the conservative management of moderate-to-large primary spontaneous pneumothorax was non-inferior to interventional management for radiological resolution at 8 weeks, with a markedly lower rate of serious adverse events [18]. Subsequent randomised work has additionally compared needle aspiration with chest-tube drainage for spontaneous pneumothorax, suggesting that simpler approaches can be effective in selected patients [19]. Reviews of ambulatory devices and small-bore catheter approaches similarly indicate that the choice of drainage technique can influence the length of stay and patient acceptability [16,17]. In trauma populations, comparative evidence indicates broadly similar outcomes between small-bore pigtail catheters and standard chest tubes [20]. Against this backdrop, the higher rate of drainage among older patients in our cohort most plausibly reflects the higher prevalence of secondary spontaneous and iatrogenic pneumothorax in older age groups than an age-driven choice of intervention per se [11,13].

A further point of clarification concerns the relationship between nursing-care dependency at admission and treatment modality. The original manuscript reported a statistically significant association on the four-tier dependency scale (χ^2^ *p* = 0.038). The re-examination of the same data using the clinically meaningful dichotomy of high (Categories 3–4) vs. low (Categories 1–2) dependency, with Haldane–Anscombe-corrected Fisher exact testing, no longer supports an independent association (OR 0.39; 95% CI 0.12–1.26; *p* = 0.193). The original four-level analysis is undermined by the fact that 37.5% of cells had expected counts below 5, which violates the conditions for stable χ^2^ inference; the dichotomised Fisher exact estimate is therefore more appropriate for the available sample. The previously reported association should accordingly be treated as a spurious finding arising from sparse-cell sensitivity rather than as a clinically meaningful effect.

### 4.3. Nursing Care

Our nursing-care findings extend the clinical description of pneumothorax in three important ways. First, the frequency profile of NANDA-I diagnoses—dominated by risk-oriented diagnoses (Risk for falls, Risk for pressure ulcers, and Risk for infection) and self-care deficits (Bathing, Feeding, and Toileting)—is consistent with the small but growing body of nursing literature describing care priorities in patients with chest drainage, in which the surveillance of the drainage system, infection prevention, mobilisation, and patient education are repeatedly identified as central nursing responsibilities [20,21]. Our results add empirical, denominator-based frequency data with explicit 95% Wilson confidence intervals to this largely descriptive literature [20]. Second, the prevalence of the evidence-based knowledge of chest-tube management among intensive-care nurses has been shown to be variable in international cohorts, suggesting that the structured documentation of nursing diagnoses—as we have undertaken here—may also serve as an indirect indicator of practice consistency [21].

Third, the bivariate analyses of the five most prevalent diagnoses identified clinically coherent patterns. Risk for falls was substantially more common in older patients and in those whose dependency category deteriorated during admission, in keeping with falls being a multifactorial outcome of cumulative frailty, polypharmacy, and reduced functional reserve. Risk for pressure ulcers, in turn, was associated with both a prolonged length of stay and high dependency at admission, consistent with the well-established role of immobility and dependence on nursing care in pressure-injury risk. At the patient level, older patients, patients with a prolonged length of stay, and patients with a high or deteriorating dependency consistently carried more NANDA-I diagnoses than their counterparts, supporting the use of the total diagnosis count as a summary indicator of nursing-care complexity.

The dependency-category data deserve particular comment. Although the overall shift in dependency between admission and discharge did not reach statistical significance on Wilcoxon signed-rank testing, 23.3% of patients deteriorated by at least one category, indicating that the post-acute nursing-care needs of this cohort are not negligible. After adjustment in the L1-penalised model, age ≥ 60 years was the only independent correlate of functional deterioration (adjusted OR 4.29; 95% CI 1.21–22.62; *p* = 0.028), with no independent effect of sex, chest drainage, or prolonged length of stay. This finding is broadly consistent with previous reports that nursing-care dependency is an important determinant of workload and resource use, with measurable variation across clinical and surgical inpatient units and across diagnostic groups [23,24,25]. The conceptual basis for measuring nursing dependency in relation to clinical case-mix and resource use was articulated in the early work on nursing dependency, diagnosis-related groups, and length of hospital stay [36], and the methodological rigour of more recent patient-classification systems has been demonstrated through follow-up studies of their reliability and validity in everyday nursing practice [32]. Comparable instruments have also been used to inform the dimensioning of nursing staff across inpatient services [33].

Finally, our use of the NANDA-I classification, in its 2024–2026 edition, provides a standardised vocabulary that facilitates the international comparability of nursing-care data [22]. We acknowledge that other standardised nursing terminologies (e.g., the International Classification for Nursing Practice, ICNP^®^) are also in use internationally and that the choice of terminology can influence both the visibility and the comparability of nursing-sensitive outcomes [34].

### 4.4. Limitations

Several limitations of this study should be acknowledged. First, the single-centre retrospective design limits the external validity of our findings and precludes inferences about the wider Croatian or central-European pneumothorax population. Second, the modest sample size (n = 60) substantially restricted the statistical power available to detect small-to-moderate effect sizes; the post hoc power calculation indicates that the study was sensitive only to large effects (Cramér’s V ≥ 0.36) in 2 × 2 contingency tables, and non-significant findings should not be interpreted as evidence of no association. Third, the modest number of events per variable in the multivariable models for prolonged length of stay (3.2 events per variable) and worsening of dependency category (3.5 events per variable) is below the conventional benchmark of 10 events per variable for unpenalised logistic regression; we used L1 penalisation and bootstrap inference to attenuate this limitation [30,31], but the adjusted estimates should nonetheless be regarded as hypothesis-generating rather than as definitive. Fourth, the use of categorical age and length-of-stay bands, dictated by the design of the original data-collection instrument, precluded continuous-variable analyses that would have been more powerful and limited the granularity of the effect-size estimates. Fifth, contemporary management guidelines [14,15] and reviews of ambulatory devices [16,17] have continued to evolve during and after our study period, and treatment patterns at the institution may already have shifted towards more conservative or ambulatory-based approaches; the present descriptive baseline may therefore not fully capture the current practice. Sixth, the four-tier Croatian dependency scale used routinely in our hospital has not been formally re-validated against international patient-classification systems [23,25], although it is conceptually comparable. Finally, important clinical covariates—including smoking status, body mass index, the size of the pneumothorax on imaging, and the presence of underlying lung disease—were not consistently recorded in the source documentation and could not be entered into the multivariable models; this constitutes a meaningful source of residual confounding.

### 4.5. Implications for Clinical Practice

Despite these limitations, the present study has practical implications for clinical and nursing practice at our institution and, by extension, at comparable secondary-care centres. The systematic documentation of NANDA-I nursing diagnoses [22], combined with the structured recording of dependency category at admission and discharge, provides a feasible framework for capturing nursing-sensitive aspects of the pneumothorax care pathway. The high prevalence of risk-oriented diagnoses such as Risk for falls and Risk for pressure ulcers—and their measurable association with older age, prolonged length of stay, and high dependency—suggests that risk-assessment protocols and targeted preventive interventions should be applied routinely from admission, particularly in older patients undergoing prolonged chest drainage [1,7,11]. The non-negligible proportion of patients who deteriorated functionally by discharge highlights the need for early-mobilisation strategies and structured discharge planning, ideally with an explicit handover to community nursing services where applicable [21].

### 4.6. Future Research

Future studies should extend the present descriptive baseline in three directions. First, multicentre cohort studies, ideally with prospective data collection, would provide more robust estimates of the population-level incidence of pneumothorax in Croatia and would permit a meaningful comparison with international cohorts [8,11]. Second, an evaluation of the comparative effectiveness of chest drainage, ambulatory small-bore drainage, and conservative management, stratified by aetiological category and patient age, would help to clarify the optimal first-line treatment for older patients with secondary spontaneous and iatrogenic pneumothorax [13,18]. Third, the integration of structured NANDA-I documentation and validated dependency instruments into routine pneumothorax pathways could support the development of nursing-sensitive quality indicators specific to chest-drainage care and inform staffing models for thoracic-surgical and respiratory units [20,24,25].

## 5. Conclusions

Patients hospitalised with pneumothorax at our regional Croatian centre over a three-year period were predominantly older men, and almost three quarters received chest drainage as their primary treatment. After adjustment for sex and nursing dependency in an L1-penalised model, age ≥ 60 years emerged as the only covariate independently associated with the receipt of chest drainage, with a prolonged length of stay and with a worsening of the dependency category between admission and discharge. The previously reported association between dependency at admission and treatment modality was not robust to dichotomisation at the clinically meaningful high-versus-low threshold and is best regarded as a sparse-data artefact rather than as a clinical signal. The nursing-care burden was substantial: most patients carried at least one NANDA-I nursing diagnosis, risk-oriented diagnoses predominated, and almost one in four patients deteriorated by at least one dependency category between admission and discharge. Given the modest sample size, the single-centre retrospective design, and the limited number of events per variable in the multivariable models, all adjusted estimates should be interpreted as hypothesis-generating. The present findings nonetheless provide a useful baseline for the Croatian setting and outline testable questions for larger, prospective, multicentre studies that integrate clinical, radiological, and nursing-sensitive endpoints.

## Figures and Tables

**Table 1:** Sex and age distribution of the study population (n = 60).

Characteristic	n	%
Sex		
Male	39	65.0
Female	21	35.0
Age (years)		
<18	2	3.3
18–29	12	20.0
30–39	4	6.7
40–49	3	5.0
50–59	6	10.0
≥60	33	55.0

Percentages refer to the total study population (n = 60).

**Table 2:** Pooled descriptive characteristics with 95% Wilson score confidence intervals (n = 60).

Characteristic	n (%)	95% CI (%)
Male sex	39 (65.0)	52.4–75.8
Age ≥ 60 years	33 (55.0)	42.5–66.9
Pneumothorax type—spontaneous	27 (45.0)	33.1–57.5
Pneumothorax type—traumatic	23 (38.3)	27.1–51.0
Pneumothorax type—iatrogenic	10 (16.7)	9.3–28.0
Treatment with chest drainage	44 (73.3)	61.0–82.9
Length of stay > 14 days	13 (21.7)	13.1–33.6
Dependency category 3 or 4 at admission	17 (28.3)	18.5–40.8
Worsening of dependency category during admission	14 (23.3)	14.4–35.4

Confidence intervals (CI) for proportions were calculated using the Wilson score method [32].

**Table 3:** Length-of-stay bands across pneumothorax type (n = 60).

Length of Stay (days)	Spontaneous (n = 27)	Traumatic (n = 23)	Iatrogenic (n = 10)
1–7	13 (48.1)	10 (43.5)	2 (20.0)
8–14	10 (37.0)	8 (34.8)	4 (40.0)
>14	4 (14.8)	5 (21.7)	4 (40.0)

Cell entries are n (% within column). Overall Fisher–Freeman–Halton *p* = 0.462.

**Table 4:** Bivariate predictors of prolonged length of stay (>14 days; 13 events) and worsening of dependency category (14 events) (n = 60).

Predictor	OR (95% CI) ^1^	*p*-Value ^2^
Outcome: length of stay > 14 days		
Age ≥ 60 vs. <60 years	5.21 (1.19–22.91)	0.026
Male vs. female sex	1.88 (0.49–7.20)	0.512
Chest drainage vs. conservative management	1.99 (0.44–8.92)	0.481
Dependency category 3–4 vs. 1–2 at admission	2.75 (0.79–9.52)	0.163
Worsening of dependency category	2.62 (0.72–9.49)	0.159
Outcome: worsening of dependency category		
Age ≥ 60 vs. <60 years	5.93 (1.36–25.90)	0.013
Male vs. female sex	2.13 (0.56–8.08)	0.340
Chest drainage vs. conservative management	1.32 (0.34–5.12)	0.740
High dependency (3–4) at admission	0.41 (0.09–1.80)	0.310
Length of stay > 14 days	2.62 (0.72–9.49)	0.159

^1^ Odds ratios with Haldane–Anscombe continuity correction (0.5 added to each cell of the 2 × 2 table). ^2^ Fisher exact *p*-values (two-sided).

**Table 5:** L1-penalised multivariable logistic regression (α = 0.5) for three nursing-sensitive outcomes; 95% confidence intervals and *p*-values from 1000-iteration non-parametric bootstrap (n = 60).

Covariate	Adjusted OR (95% CI)	*p*-Value
Outcome: receipt of chest drainage (44 events)		
Age ≥ 60 years	3.67 (1.21–13.56)	0.026
Male sex	2.82 (1.00–10.05)	0.100
Dependency category 3–4 at admission	0.46 (0.10–1.06)	0.240
Outcome: length of stay > 14 days (13 events)		
Age ≥ 60 years	3.69 (1.02–21.00)	0.042
Male sex	1.57 (0.75–6.15)	0.574
Chest drainage	1.00 (0.43–4.13)	1.000
Dependency category 3–4 at admission	2.26 (0.78–9.92)	0.314
Outcome: worsening of dependency category (14 events)		
Age ≥ 60 years	4.29 (1.21–22.62)	0.028
Male sex	1.60 (0.70–7.46)	0.508
Chest drainage	0.81 (0.17–1.69)	0.792
Length of stay > 14 days	1.21 (0.46–6.07)	0.752

Models were fitted by L1-penalised (lasso-type) logistic regression with penalty α = 0.5. 95% CIs and *p*-values were obtained from 1000 non-parametric bootstrap replications [33,34]. Covariates were selected on clinical grounds; type of pneumothorax was excluded from the model for receipt of chest drainage because of complete separation in the iatrogenic subgroup.

**Table 6:** Frequency of all documented NANDA-I nursing diagnoses with 95% Wilson confidence intervals (n = 60).

NANDA-I Code	Nursing Diagnosis	n (%)	95% CI (%)
Five most prevalent diagnoses			
00155	Risk for falls	35 (58.3)	45.7–69.9
00108	Bathing self-care deficit	16 (26.7)	17.1–39.0
00249	Risk for pressure ulcers	11 (18.3)	10.6–29.9
00004	Risk for infection	8 (13.3)	6.9–24.2
00092	Activity intolerance	7 (11.7)	5.8–22.2
Other diagnoses			
00146	Anxiety	4 (6.7)	2.6–15.9
00126	Deficient knowledge	4 (6.7)	2.6–15.9
00030	Impaired gas exchange	4 (6.7)	2.6–15.9
00102	Feeding self-care deficit	4 (6.7)	2.6–15.9
00148	Fear	4 (6.7)	2.6–15.9
00132	Acute pain	3 (5.0)	1.7–13.7
00110	Toileting self-care deficit	3 (5.0)	1.7–13.7
00248	Impaired tissue integrity ^1^	2 (3.3)	0.9–11.4
00032	Ineffective breathing pattern	2 (3.3)	0.9–11.4
00109	Dressing self-care deficit	2 (3.3)	0.9–11.4
00031	Ineffective airway clearance	2 (3.3)	0.9–11.4
00094	Risk for activity intolerance	2 (3.3)	0.9–11.4
00130	Disturbed thought process	1 (1.7)	0.3–8.9
00039	Risk for aspiration	1 (1.7)	0.3–8.9

^1^ Documented as decubitus ulcer. Percentages refer to the total study population (n = 60). A single patient could contribute more than one diagnosis; a total of 116 diagnostic episodes were recorded across 56 patients. Four patients (6.7%) had no diagnosis recorded.

**Table 7:** Bivariate associations between the five most prevalent NANDA-I nursing diagnoses and pre-specified covariates (n = 60).

Diagnosis (NANDA-I Code)	Characteristic	OR (95% CI) ^1^	*p*-Value ^2^
Risk for falls (00155)	Age ≥ 60 vs. <60 years	5.15 (1.54–18.91)	0.004
	Male vs. female sex	1.08 (0.32–3.58)	1.000
	Chest drainage vs. conservative	1.58 (0.43–5.87)	0.556
	Length of stay > 14 vs. ≤14 days	5.14 (0.96–52.74)	0.054
	Dependency 3–4 vs. 1–2 at admission	3.05 (0.77–14.92)	0.089
	Worsening of dependency category	5.84 (1.11–59.53)	0.028
Bathing self-care deficit (00108)	Age ≥ 60 vs. <60 years	0.54 (0.14–1.99)	0.382
	Male vs. female sex	0.61 (0.16–2.34)	0.541
	Chest drainage vs. conservative	0.34 (0.08–1.38)	0.100
	Length of stay > 14 vs. ≤14 days	3.10 (0.69–13.82)	0.088
	Dependency 3–4 vs. 1–2 at admission	2.60 (0.65–10.39)	0.193
	Worsening of dependency category	1.13 (0.22–4.94)	1.000
Risk for pressure ulcers (00249)	Age ≥ 60 vs. <60 years	2.52 (0.52–16.51)	0.315
	Male vs. female sex	1.54 (0.32–10.14)	0.731
	Chest drainage vs. conservative	0.96 (0.19–6.49)	1.000
	Length of stay > 14 vs. ≤14 days	6.87 (1.35–37.97)	0.009
	Dependency 3–4 vs. 1–2 at admission	6.55 (1.36–37.07)	0.008
	Worsening of dependency category	3.61 (0.71–18.14)	0.107
Risk for infection (00004)	Age ≥ 60 vs. <60 years	2.73 (0.44–30.14)	0.276
	Male vs. female sex	1.71 (0.27–19.00)	0.701
	Chest drainage vs. conservative	— ^3^	0.095
	Length of stay > 14 vs. ≤14 days	2.47 (0.33–15.41)	0.353
	Dependency 3–4 vs. 1–2 at admission	— ^3^	0.091
	Worsening of dependency category	2.20 (0.30–13.52)	0.374
Activity intolerance (00092)	Age ≥ 60 vs. <60 years	2.20 (0.32–25.10)	0.442
	Male vs. female sex	— ^3^	0.085
	Chest drainage vs. conservative	2.34 (0.25–116.10)	0.663
	Length of stay > 14 vs. ≤14 days	3.15 (0.40–22.09)	0.166
	Dependency 3–4 vs. 1–2 at admission	1.01 (0.09–7.07)	1.000
	Worsening of dependency category	1.36 (0.12–9.70)	0.660

^1^ Odds ratios with Haldane–Anscombe continuity correction (0.5 added to each cell). ^2^ Two-sided Fisher exact *p*-values. ^3^ OR not estimable because of a structural zero cell.

**Table 8:** Distribution of the total number of documented NANDA-I nursing diagnoses per patient across pre-specified strata (n = 60).

Characteristic	Median (IQR) per Group	*p*-Value ^1^
Age ≥ 60 vs. <60 years	2 (1–3) vs. 1 (1–2)	0.006
Male vs. female sex	2 (1–3) vs. 1 (1–3)	0.424
Chest drainage vs. conservative management	2 (1–3) vs. 1 (1–3)	0.794
Length of stay > 14 vs. ≤14 days	2 (2–3) vs. 1 (1–2)	0.001
Dependency category 3–4 vs. 1–2 at admission	2 (1–3) vs. 2 (1–2)	0.048
Worsening of dependency category vs. none	2 (2–3) vs. 2 (1–2)	0.048
Type of pneumothorax (3-level): Sp/Tr/Iat	1 (1–3)/2 (1–2)/2 (2–3)	0.662 ^2^
Dependency category 1/2/3/4 at admission	1/2/2/4	0.015 ^2^

^1^ Mann–Whitney U test for two-group comparisons. ^2^ Kruskal–Wallis’s test for comparisons across three or more groups.

## Data Availability

The aggregated data underlying the analyses in this article are contained within the article. The de-identified individual-level dataset can be made available by the corresponding author upon reasonable request, subject to the institutional data-protection policies of Varaždin General Hospital.

## Abbreviations

The following abbreviations are used in this manuscript:

aOR	adjusted odds ratio
BIS	Bolnički informacijski sustav (Hospital Information System)
CI	confidence interval
COPD	chronic obstructive pulmonary disease
ICNP	International Classification for Nursing Practice
ICU	intensive care unit
IQR	interquartile range
LOS	length of stay
NANDA-I	NANDA International
OR	odds ratio
SD	standard deviation
STROBE	Strengthening the Reporting of Observational Studies in Epidemiology
